# A human-scale perspective on global warming: Zero emission year and personal quotas

**DOI:** 10.1371/journal.pone.0179705

**Published:** 2017-06-19

**Authors:** Alberto de la Fuente, Maisa Rojas, Claudia Mac Lean

**Affiliations:** 1 Civil Engineering Department, Universidad de Chile, Santiago, Chile; 2 Geophysics Department, Universidad de Chile, Santiago, Chile; 3 Center for Climate and Resilience Research, Universidad de Chile, Santiago, Chile; 4 Office of Engineering for Sustainable Development, Universidad de Chile, Santiago, Chile; Universidade de Vigo, SPAIN

## Abstract

This article builds on the premise that human consumption of goods, food and transport are the ultimate drivers of climate change. However, the nature of the climate change problem (well described as a tragedy of the commons) makes it difficult for individuals to recognise their personal duty to implement behavioural changes to reduce greenhouse gas emissions. Consequently, this article aims to analyse the climate change issue from a human-scale perspective, in which each of us has a clearly defined personal quota of CO_2_ emissions that limits our activity and there is a finite time during which CO_2_ emissions must be eliminated to achieve the “well below 2°C” warming limit set by the Paris Agreement of 2015 (COP21). Thus, this work’s primary contribution is to connect an equal per capita fairness approach to a global carbon budget, linking personal levels with planetary levels. Here, we show that a personal quota of 5.0 tons of CO_2_ yr^-1^ p^-1^ is a representative value for both past and future emissions; for this level of a constant per-capita emissions and without considering any mitigation, the global accumulated emissions compatible with the “well below 2°C” and 2°C targets will be exhausted by 2030 and 2050, respectively. These are references years that provide an order of magnitude of the time that is left to reverse the global warming trend. More realistic scenarios that consider a smooth transition toward a zero-emission world show that the global accumulated emissions compatible with the “well below 2°C” and 2°C targets will be exhausted by 2040 and 2080, respectively. Implications of this paper include a return to personal responsibility following equity principles among individuals, and a definition of boundaries to the personal emissions of CO_2_.

## Introduction

Recent studies have shown that global warming in a single year is nearly proportional to the total cumulative amount of CO_2_ emitted since the pre-industrial period [1–3]. The reason for this relationship is both that the radiative forcing of CO_2_ is larger than those of any other greenhouse gas and that the removal processes of CO_2_ from the atmosphere require a long time scale [4]. As a result, to achieve the “well below 2°C” warming limit set in the Paris Agreement of 2015 (COP21) [5], the amount of cumulative CO_2_ emissions since the pre-industrial era is limited, and anthropogenic CO_2_ emissions eventually must cease [2–4,6–8].

Since early attempts to halt global warming, the political discussion has adopted a top-down approach focused on countries that should reduce their emissions [9–12]. Pursuant to this top-down approach, universal agreement has been partially blocked by the different perspectives of industrialised developed countries with high per-capita emissions that are historically responsible for most of the emissions, and populous developing countries with larger growth rates in per-capita emissions [9–11,13]. Recent alternatives have proposed focalising cuts in CO_2_ emissions in a few leader countries as a sort of national voluntary scheme [14]. However, human consumption of goods, food and transportation are the ultimate drivers of climate change [15], and we argue that the top-down approach underestimates this fact such that together with worldwide agreements and technological upgrades, changes in human behaviour are critical to address climate change.

Part of the success of the COP21 and the rapid enforcement of the Paris Agreement can be explained by the different approach adopted in the Paris negotiations. At the UN Climate Change Conference in Durban (COP17, 2011), all countries recognised that a new universal, legal agreement needed to be developed in which all countries had shared but differential duties. This conviction materialised in the Intended National Determined Contributions (INDCs) to reduce greenhouse gas emissions that most countries delivered ahead of the COP21. Allowing each country to draft their INDCs independently, taking into account their own national circumstances, gave them the ownership that was required for the adoption of the Paris Agreement. This success highlights the importance of a “bottom-up” approach to solve the global climate change problem. Despite the success of bottom-up contributions at the country level, we argue that engagement in fighting climate change at the individual level (and therefore in human behaviour) are critical to address climate change effectively [16,17]. Part of the “grasping” problem that prevents humans from feeling responsible for the global climate change problem is related to the nature of the issue [18]. Well described as a tragedy of the commons [19], climate change is produced by the collective action of billions of individuals—many of whom are already dead, although the costs of their actions are shared among the entire population, including many not yet born. In other words, actions and consequences in the context of climate change are disjointed in both space and time, which makes it very difficult for individuals to engage in actions against global warming [18]. The personal engagement required is further inhibited by the fact that for most people, the environment alone is not a real motivation [20]. Although civic engagement is more related to human rights, community or the impact on poorer people [20], it can be catalysed by personal experiences such as carpooling or recycling [21]. In addition, if people do not feel or see that they are personally affected by climate change, it will be more difficult to achieve the required changes [22]. Likewise, as the number of individuals who perceive changes in climate increases over time, it can be argued that the number of people who will perform specific actions against climate change will also increase [21,23]. Finally, understanding that intentions are precursors of actions, then the formulation and promotion of a specific intention would require a clear definition of the target, the situation and the time frame [20,24]. In this sense, we argue that important information and efforts required to delineate this target and time period, is still insufficient. More specifically, we propose that the definition of a personal quota of CO_2_ emissions, and the estimated year in which all anthropogenic CO_2_ emissions should stop (hereinafter called the zero emission year, *ZEY*), can help fill this gap. By determining the order of magnitude of these two parameters, a personal limit regarding emissions can be set on human activity, and each person will have the required information to evaluate his or her own personal actions against climate change, in what is called the human-scale perspective that follows the ideas previously described for economic development and human needs [25]. The expected impact of adopting a human-scale perspective on climate change is to return the responsibility to the individual level. The proposal to define a uniform personal quota does not ignore the “common but differentiated responsibility principle” [26] upon which climate negotiations build, but is rather a first-order, idealised approximation of the goal of introducing a human-scale perspective, thereby allowing humans to “grasp” the climate change problem while connecting it to the global situation. The definition of a personal quota of CO_2_ emissions is not novel as an alternative to allocate emissions among countries [27,28]; it constitutes a burden sharing option that is strongly based on equity principles among humans, without any type of differentiation between them. In this manner, our definition of the *ZEY* extends previous research [29], connecting personal behaviour with the planetary carbon budget and boundaries, by defining a uniform per capita fairness approach to a global carbon budget with an associated timeframe. Initially, we share the definition of fairness, which does not differentiate between, for example, warm and cold countries [29]. Such differentiations can be included based on, for example, the greenhouse development rights framework [13]. Hence, the aim of this paper is to contribute to the discussion on the urgency of adopting greenhouse gas emissions cuts on the basis of the global carbon budget, within the burden sharing framework, by defining two easily read numbers and concepts aimed at individuals.

The full set of Coupled Modelling Intercomparison Project 5 (CMIP5) simulations [30] are used in this work. We complemented the analysis with the numerical model MAGICC [2,31,32] for validating the proposed *ZEY* and personal quota, while adopting the Paris Agreement target of “well below 2°C”.

## Methods

### Zero emission year and remaining global CO_2_ quota

**Fig 1:**
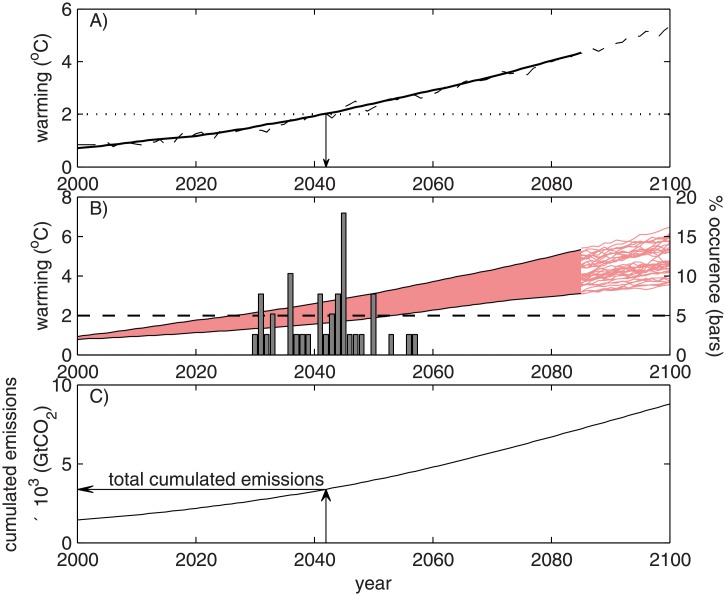
A) Time series of global warming (dashed line) simulated RCP 8.5 scenario by the model CSIRO-Mk3-6-0 of CSIRO. The solid line corresponds to the 30-year moving average, and the year when this time series crosses the 2°C threshold is defined as the zero emission year. B) Example of the frequency distribution of zero emission years for entire set of simulations of the RCP8.5 scenario. The shaded area in light red corresponds to the global climate warming in the RCP8.5 simulations, whereas the lines after 2085 correspond to the individual time series of global warming. C) The time series of cumulative emissions since the pre-industrial era and the definition of the total cumulative emissions required to avoid exceeding the 2°C warming limit for the *ZEY* of 2042 defined in A).

The zero emission year is estimated as the first year in which the global mean temperature of a particular CMIP5 simulation exceeds 2°C. Time series of the global mean temperature are available in the Annex I of the fifth IPCC report [33], which numerically expresses the global temperature referring to the year 2005, when global warming since the pre-industrial era (1850–1900) was 0.61°C [33]. Each of the simulated global warming time series were smoothed with a 30-year moving average filter. An example of this definition is shown in Fig 1A, in which the dashed and solid lines correspond to the simulated global warming time series with respect to the pre-industrial era and the corresponding 30 years moving average time series, respectively, which with corresponds to the RCP 8.5 scenario simulated by the model CSIRO-Mk3-6-0. For this simulation, warming reaches 2°C in the year 2042, which corresponds to the zero emission year of that particular simulation. Overall, 107 out of 138 CMIP5 simulations indicate climate warming greater than or equal to 2°C; 7 of which correspond to the RCP2.6 scenarios, 36 to the RCP4.5 scenarios, 25 to the RCP6.0 scenarios, and 39 to the RCP8.5 scenarios. Fig 1B shows the frequency distribution of the zero emission year computed for the entire set of 39 simulations of the RCP 8.5 simulation, in which the shaded area in the light red colour defines the range of variation of climate warming time series of the RCP 8.5. Note that the CMIP5 simulations end in 2100 and that the 30 year moving average is not defined after 2085.

Each of the CMIP5 simulations has a corresponding time series of CO_2_ emissions (fossil and industrial emissions, and land use-related emissions) defined by the four RCP scenarios [30,34]. As a consequence, the cumulative CO_2_ emissions between the pre-industrial era (1850) and the zero emission year are the total cumulative emissions required to meet the warming limit of 2°C (Fig 1C). Notice that recent studies show that a sudden stop of CO_2_ emissions overestimates the remaining global quota [35,36]. This aspect is presented in the discussion section.

The remaining global quota after 2016 was estimated considering that the cumulative CO_2_ emissions between the pre-industrial era (1850) and 2015 were 2094±5% Gt CO_2_ [37]. This estimation includes the 2016 global carbon budget [37]. As a result of this estimation, a set of 107 values of the remaining global quota after 2016 were obtained, all of them equally probable. With this information, we conducted a frequency distribution analysis of the CMIP5 ensemble of opportunity associated with a particular value of the remaining global quota. The frequency distribution of the CMIP5 ensemble of opportunity [38,39], hereinafter called the ensemble of opportunity, of one of the remaining global quotas is computed as the number of simulations that have a remaining global quota larger than the value in question divided by the total number of simulations. The minimum value of the remaining global quota obtained from all of the CMIP5 simulations is used to characterise the “well below 2°C” target of COP21.

### Personal quota of CO_2_ emissions

**Fig 2:**
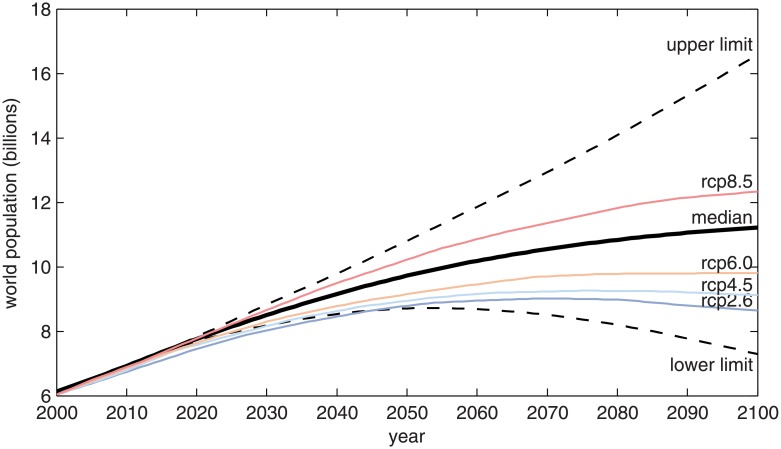
Estimated time series of the world population [34,40]. The solid black line corresponds to the median population, and the dashed lines correspond to the upper and lower limits of the projection [40]. Coloured lines correspond to time series of world population associated with the different RCP scenarios [34].

Based on the remaining global quota, the definition of the personal quota requires a definition of how this remaining global quota will be spent in the remaining time to the *ZEY* and an estimation of the human population (*W*_*p*_(*t*), Fig 2). The remaining global quota, *T*, can be related to the personal quota as follows:

$$T\;=\;\int_{2016}^{ZEY}Q\left(t\right)\;W_{p}\left(t\right)\;dt$$ (1)

where *Q* denotes the personal quota written in terms of tons of CO_2_ yr^-1^ p^-1^ and *W*_*p*_ is the world populationWe use the median world population estimated by the Population Division of the United Nations [40], which estimates the present world population in 7.3 billion and the median world population in 2030, 2045 and 2060 of 8.5, 9.45 and 10.18 billion, respectively (Fig 2).

First we studied a highly idealised and worst-case scenario that considers a constant personal quota until the *ZEY* without the implementation of any mitigation policy or technological upgrades to reduce this value. This scenario allows us to link the *ZEY* and the personal quota, and to define a baseline personal quota. Then we studied more plausible scenarios that consider a linear reduction in time of the personal quota, which depends on the year at which the reduction of *Q* starts to occur.

### Validation of *ZEY* and personal quota

To validate the definition of the *ZEY* and the personal quota, the simplified climate-carbon cycle model MAGICC [30] was used. MAGICC stands for Model for the Assessment of Greenhouse Gas Induced Climate Change and solves a coupled ocean-atmosphere system and a world average carbon cycle model. MAGICC uses as its input time series of greenhouse gases emissions and gives as a result a time series of greenhouse gas concentrations in the atmosphere and global warming with respect to the pre-industrial period. Parameters involved in the model were calibrated for each of the CMIP5 models [30], and based on historical observations, a set of 600 probabilistic/historically constrained runs were included in MAGICC for quantifying uncertainty in the analysis [2,31,32].

MAGICC simulations start in 2000, and to construct the time series of greenhouse gas emissions that forces this model, the time series of CO_2_ emissions from CDIAC (cdiac.ornl.gov, [37]) were used for the period between 2000 and 2015. Different scenarios of CO_2_ emissions between 2016 and 2100 are built based on a uniform personal quota, and the time series of the median world population estimated by the United Nations [40]. With respect to the emissions of other greenhouse gasses, CDIAC information shows that emissions since 2005 are well represented by the RCP8.5 scenario. Consequently, the time series of emissions of other greenhouse gases in the RCP8.5 scenario was used for the period 2005–2015 in all of the simulations. Between 2016 and 2020, a linear transition between emissions of RCP8.5 and RCP2.6 is considered, and after 2020 the time series of emissions of other greenhouse gases of the RCP2.6 scenario was used. This is because the RCP2.6 scenario is the only scenario that is coherent with the 2°C target for warming. The following scenarios are considered for the CO_2_ emissions as a function of representative values of the remaining global quota *T*:

- *T* = ∞. This is the base simulation, in which CO_2_ emissions constantly increase in time as the world population increases, considering a uniform personal quota. The time series of the median world population estimated by [40] is used for the calculation. This base simulation also considers a linear transition between the present per-capita emissions (5.59 tons of CO_2_ yr^-1^ p^-1^, [37]) and the personal quota that is reached in 2020.
- *T* for 2°C. For this target, we use the following definition: this simulation follows the pathway of *T* = ∞ simulation and suddenly stops CO_2_ emissions at the *ZEY* required to meet remaining global quota associated to the 2°C warming limit with 50% of the ensemble of opportunity. Based on this simulation, subsets of simulations were defined to study the influence of a gradual reduction in time of the personal quota. These simulations consider a constant and uniform personal quota until a certain year (2020, 2025, etc.) and then a linear reduction in the per-capita emissions in such a way that the total accumulated CO_2_ emissions are the same for the base simulation that suddenly stops the CO_2_ emissions.
- *T* for “well below 2°C” target. For this target, we use the following definition: this simulation follows the pathway of *T* = ∞ simulation and suddenly stops CO_2_ emissions at the *ZEY* required for the minimum remaining global quota, i.e., the remaining global quota for which 100% of the CMIP5 simulations reach 2°C. The same type of subsets of simulations with a gradual reduction in time of the personal quota is defined for this “well below 2°C” target simulation. These sets of simulations are coherent with the “well below 2°C” target defined at the COP21 in Paris.

The model MAGICC uses as its input a single text file that contains the time series of greenhouse gas emissions, with annual resolution. Input files of the simulations of this article are available in S1 Appendix.

### Transformation from CO_2e_ and CO_2_

**Fig 3:**
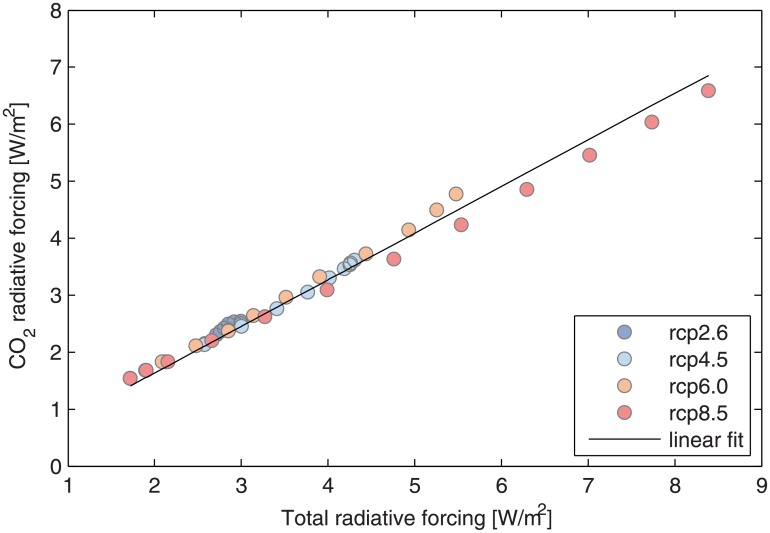
Linear fit between total radiative forcing and CO_2_ radiative forcing using information from [4].

The premise of this article is that human consumption of goods, food and transportation are the ultimate drivers of climate change. Consequently, changes in personal behaviour have a large impact on CO_2_ emissions. With this, carbon footprint calculators can now have a different reading, for example, by allowing individuals to manage the personal carbon budget by summing up their annual emissions and then to evaluate the effectiveness of a particular action. The first limitation is that carbon footprint calculators are usually written in terms of an equivalent mass of CO_2_, hereinafter called CO_2e_ (e.g., [41]), whereas the personal quota estimated here is expressed in terms of CO_2_ emissions of fossil fuels, cement, and land use-related emissions. In the absence of detailed research, the bulk transformation from CO_2e_ to CO_2_ was made based on a linear fit between the specific contributions of CO_2_ radiative forcing with respect to total radiative forcing using RCP scenarios [4]. A bulk transformation factor of 0.82±0.01 kg CO_2_/kg CO_2e_ (r^2^ = 0.998) was obtained, and the goodness of this fit is shown in Fig 3.

## Results

### Remaining global quota and zero emission year

**Fig 4:**
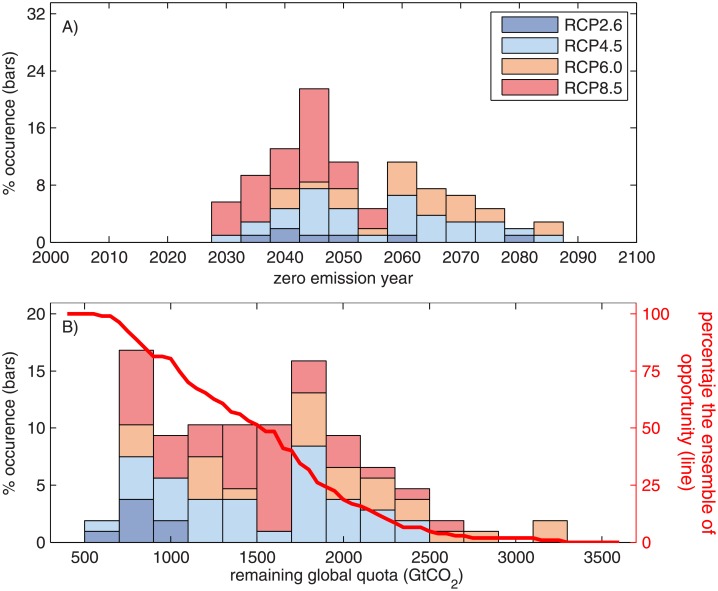
A) Frequency distribution of zero emission year. Bars indicate the percentage of the CMIP5 simulations that predicted climate warming greater than or equal to 2°C in the range of zero emission years defined by the bar width, which is 5 years. B) The frequency distribution of remaining global quota (bars), with a similar interpretation to (A), and the ensemble of opportunity (red line, right-hand side vertical axis). The probability of success of one remaining global quota was computed as the number of CMIP5 simulations with a remaining global quota greater than or equal to the remaining global quota.

The *ZEY* is the year in which CO_2_ emissions should stop to avoid global warming greater than 2°C with respect to the pre-industrial era. By using the full set of CMIP5 simulations for the four RCP emissions scenarios [34], a distribution of the *ZEY* and thus the remaining global quota can be obtained, by finding the year in which each individual simulation reaches the 2°C warming limit and relating this year with accumulated CO_2_ emissions. Therefore, the value is not unique and the obtained distribution is shown in Fig 4A, which shows that the RCP8.5 simulations are associated with closer zero emission years, whereas simulations of the RCP6.0 scenario provide values of the *ZEY* furthest away in this century. The median *ZEY* for scenarios RCP2.6, 4.5, 6.0 and 8.5 are 2043 (2037–2058), 2058 (2042–2069), 2062 (2047–2074), and 2043 (2033–2048), respectively. Hereinafter, numbers in brackets correspond to 84% and 16% of the ensemble of opportunity, respectively, representing the standard deviation and a 68% confidence interval. Similarly, the remaining global quota is not unique and its distribution is shown in Fig 4B, which takes into account the different climate sensitivity of the CMIP5 simulations. The median remaining global quota is 1562 Gt CO_2_ (894–2061). The solid line in Fig 4B shows the ensemble of opportunity for reaching maximum warming smaller than 2°C as a function of the remaining global quota. The ensemble of opportunity for one remaining global quota is the number of simulations that have a remaining global quota greater than or equal to this value divided by the total number of simulations. The maximum remaining global quota was 3288 Gt CO_2_, and the minimum was 594 Gt CO_2_. Here, the minimum remaining global quota of 594 Gt CO_2_ is related to the total amount of CO_2_ emissions required to achieve the “well below 2°C” target. As a reference, previous studies estimated the remaining global quota since 2014 at 1500 Gt CO_2_ [3], 1440 Gt CO_2_ [1], and 1620 Gt CO_2_ [36].

**Fig 5:**
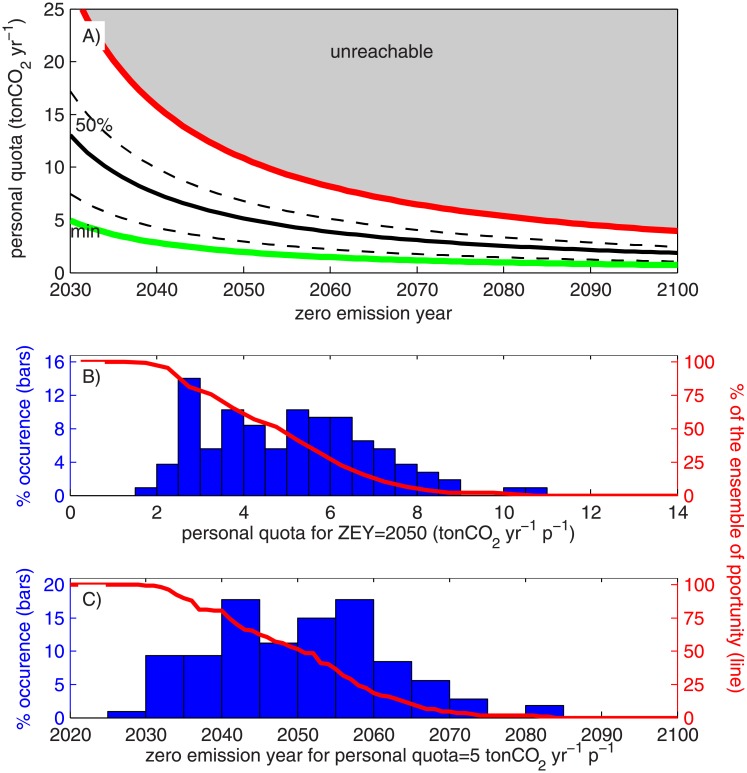
A) Curves of personal quotas as a function of the zero emission year. Each curve is associated with one percentage of the ensemble of opportunity defined for the remaining global quota. The red line defines the possible limit defined by the CMIP5 simulation with a larger remaining global quota, and green line the relationship between *ZEY* and personal quota for the minimum remaining global quota associated with the “well below 2°C” target (*T* = 594 Gt CO_2_). B) The frequency distribution of personal quotas obtained by defining a zero emission year of 2050. C) The frequency distribution of the zero emission year obtained by defining a personal quota as 5.0 tons of CO_2_ yr^-1^ p^-1^.

The curves of Fig 5A relate the constant personal quota and the *ZEY* for the 2°C target warming limit required to meet the remaining global quota associated with a given percentage of the ensemble of opportunity (red line in Fig 4B). The red line defines the limit of possible solutions given by the maximum remaining global quota estimated in Fig 4B, whereas the green curve shows the minimum remaining global quota associated with the “well below 2°C” target (*T* = 594 Gt CO_2_). Fig 5A shows that the personal quota defines the rate at which CO_2_ will be emitted. Similar to what has been recently shown [35,36], if per-capita emissions are larger, the *ZEY* will arrive sooner than for a smaller personal quota. Furthermore, Fig 5A can be used to quantify uncertainty in the value of the remaining global quota *T* in two different ways. The first way considers fixing the zero emission year, and the percentage of the ensemble of opportunity of *T* is transferred to the value of a constant personal quota. As a result, it is possible to obtain the distribution of the constant personal quota required by defining, for example, 2050 as the *ZEY* (Fig 5B). The second way of quantifying uncertainty is by fixing the value of a constant personal quota such that the *ZEY* can be associated with a percentage of the ensemble of opportunity of *T*. The result obtained by defining a constant personal quota of 5.0 tons of CO_2_ yr^-1^ p^-1^ is shown in Fig 5C, and indicates that for the median remaining global quota (*T* = 1562 Gt CO_2_) the corresponding *ZEY* is 2051, with a minimum value of 2030 associated with the “well below 2°C” target (*T* = 594 Gt CO_2_).

**Fig 6:**
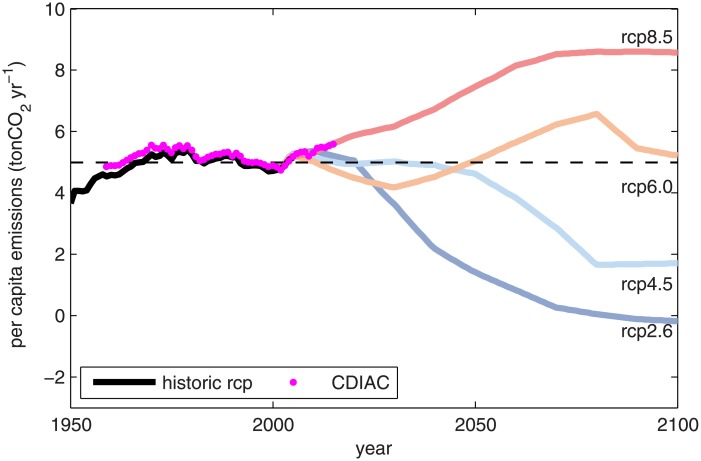
The time series of per capita emissions associated with RCP simulations (colour lines), historical per capita emission of RCP simulations (black line), and information published CDIAC (magenta circles). The dashed line corresponds to the personal quota of 5.0 tons of CO_2_ yr^-1^ p^-1^.

Given that the personal quota and the *ZEY* are complementary quantities, the final recommendation related to the personal quota is more likely to involve a socio-political definition than a scientific computation. For this definition, we looked at the general trend of historical and projected per-capita emissions. Fig 6 shows the time series of the world average per-capita emissions published by the CDIAC (cdiac.ornl.gov, magenta circles), the historical emissions of the CMIP5, and per-capita emissions associated with the RCP scenarios and the corresponding time series of world population [40]. First, Fig 6 shows that in recent years the world has followed the RCP8.5 pathway [37], which is associated with the largest personal quota, thus explaining the earlier zero emission years obtained in Fig 4A. However, the long-term perspective, given by historical per-capita emissions estimated based on the CMIP5 information (black line Fig 6) and the CDIAC data, show that per-capita emissions have remained approximately constant since 1960, with an average value of CDIAC per capita emission equal to 5.22 tons of CO_2_ yr^-1^ p^-1^ with a standard deviation of 0.23 tons of CO_2_ yr^-1^ p^-1^. Considering these past values and CMIP5 projections, a personal quota of 5.0 tons of CO_2_ yr^-1^ p^-1^ is a representative value of both past observations and future CO_2_ projections. The corresponding *ZEY* for the median remaining global quota (*T* = 1562 Gt CO_2_) is 2051 for the target warming of 2°C, whereas the *ZEY* associated with the “well below 2°C” target is 2030. For simplicity, *ZEY* = 2050 is defined for the target warming of 2°C and 50% of the ensemble of opportunity, in case of adopting a constant personal quota.

### Validation

**Fig 7:**
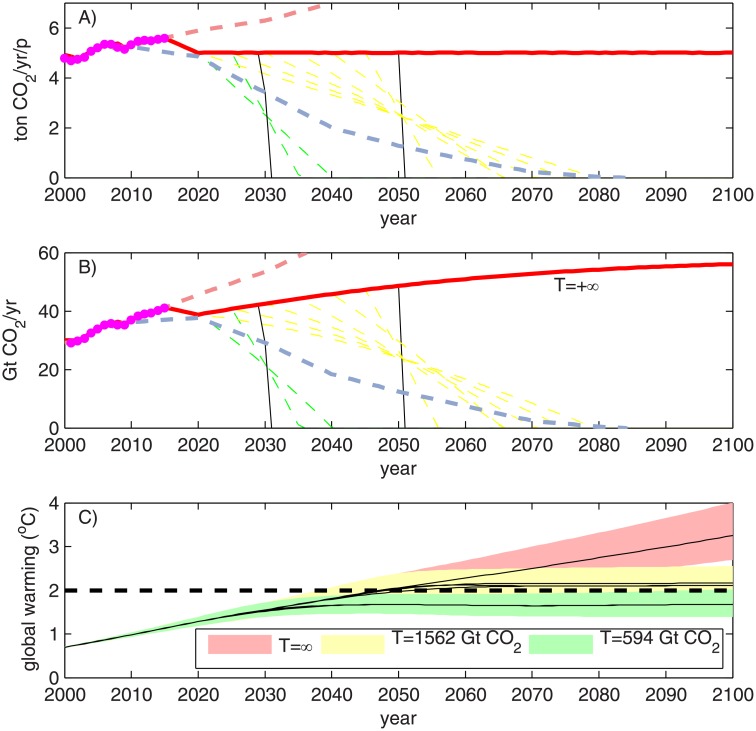
A) Time series of per capita CO_2_ emissions for the numerical model MAGICC, and scenario of *T* = ∞ (red line), constant *Q* for *T* = 1562 Gt CO2 (50% of the ensemble of opportunity) and constant *Q* for *T* = 594 Gt CO2 (“well below 2°C” target) in black lines. Subset of time series that consider a gradual reduction in per capita emissions are shown in dashed yellow (*T* = 1562 Gt CO2) and green lines (*T* = 594 Gt CO2). B) Similar to (A) for total CO_2_ emissions. Light blue and light red dashed lines in A) and B) denotes per-capita emissions and total emissions of RCP2.6 and RCP8.5 scenarios, respectively. C) Time series of global warming associated with each scenario of A and B. The coloured band denotes the standard deviation of 600 probabilistic/historically constrained runs, whereas the solid black line denotes median global warming.

Three simulations were made using the numerical model MAGICC to validate the results for the personal quota and *ZEY* associated with the “well below 2°C” and 2°C targets (*T* = 594 Gt CO_2_ and 1562, respectively). The corresponding time series of CO_2_ per capita and total CO_2_ are shown in Fig 7A and 7B, respectively, where the red line corresponds to the *T* = ∞ simulation. Black lines correspond to the simulations with a constant per capita emission until the *ZEY*; dashed lines in green and yellow are the subset of simulations that considers a constant personal quota until 2020, 2025, and then a linear reduction of *Q* as preserving the total cumulated emissions associated with 50% of the ensemble of opportunity (family of yellow dashed lines, *T* = 1562 Gt CO_2_) and the “well below 2°C” target simulation (family of green dashed lines, *T* = 594 Gt CO_2_). Fig 7A and 7B also show the time series of observed emissions (magenta dots) and CO_2_ emissions of the RCP8.5 and RCP2.6 scenarios (red dashed line and light blue dashed line).

As a response to the emissions scenarios of Fig 7A, 7B and 7C shows time series of global warming for the set of 600 probabilistic/historically constrained runs, where the black solid lines indicate the median time series and the coloured band indicates the standard deviation of the results (red for the *T* = ∞ simulation, yellow for the *T* = 1562 Gt CO_2_ simulation and green for the *T* = 594 Gt CO_2_simulation). Simulations with a gradual reduction in the per-capita emissions show no significant variation among each other, as observed in the fact that all of the median global warming values for the *T* = 594 Gt CO_2_ simulation follow essentially the same pathway, with differences of approximately 0.01°C.

As expected, the *T* = 1562 Gt CO_2_ simulations stabilise the median global warming near 2°C, thus validating the fact that the definition of a *Q* = 5.0 tons of CO_2_ yr^-1^ p^-1^ and *ZEY* = 2050 is associated with 50% of the ensemble of opportunity. Furthermore, because the standard deviation of the global warming simulated for the *Q* = 5.0 tons of CO_2_ yr^-1^ p^-1^ and *ZEY* = 2030 scenario is associated with global warming of less than 2°C, this scenario is coherent with the well below 2°C target defined in the Paris Agreement. The median global warming associated with *T* = 594 Gt CO_2_ is 1.68°C, and 87% of the runs simulated global warming of more than 1.37°C.

## Discussion

### Zero emission year

Based on the results presented in this article, we obtain that a scenario that considers a constant personal CO_2_ quota equal to 5.0 tons of CO_2_ yr^-1^ p^-1^ and *ZEY* = 2030, is consistent with the “well below 2°C” target defined at the COP21 (*T* = 594 Gt CO_2_). The “well below 2°C” target is defined with the 100% frequency distribution of the CMIP5 ensemble, reaching 2°C. Furthermore, simulations for a *T* = 1562 Gt CO_2_ show that to consider a constant personal quota equal to 5.0 tons of CO_2_ yr^-1^ p^-1^ and a *ZEY* = 2050 is a scenario consistent with the 2°C warming target with a 50% frequency distribution of the CMIP5 ensemble. We stress that the goal of this paper is not to provide a realistic mitigation pathway but instead to help translate the global climate change problem to a human-scale perspective related to our personal behaviour as citizens/consumers. In this sense, even though the constant personal quota scenario until the *ZEY* is not a realistic mitigation pathway, the *ZEYs* here defined are a concrete estimation of the timeframe available to eliminate anthropogenic CO_2_ emissions.

The gradual reduction of CO_2_ emissions in time is a more realistic mitigation pathway than a sudden stop scenario used for estimating the *ZEY*. If this is the case, the personal quota of 5.0 tonCO_2_ yr^-1^ p^-1^ should be understood as the personal quota for the next few years, which must be reduced in time to achieve the transition towards a carbon neutral world. Here it is important to remark that the 2016 per capita emission is 5.59 tonCO_2_ yr^-1^ p^-1^, such that the personal quota of 5.0 tonCO_2_ yr^-1^ p^-1^ requires a 10% reduction in today’s per capita emissions. Similar results showed that CO_2_ emissions should be eliminated by 2070 for a medium chance (frequency distribution between 50% and 66%) of limiting global warming below 2°C; and 2050 for a medium chance of limiting global warming below 1.5°C [35]. It is important to highlight that the sooner world average per-capita emissions start to decrease, the longer the available time to eliminate net CO_2_ emissions (Fig 7A).

Furthermore, despite the simplified assumptions used to calculate the *ZEY*, it clearly identifies the human generation who will be primarily responsible for the transition towards eliminating anthropogenic CO_2_ emissions. Climate change has been usually considered as a transgenerational problem; however, the *ZEYs* of 2030 and 2050 obtained for a constant personal quota show that the solution does not depend on future generations, indicating the urgency of implementing far-reaching solutions for eliminating CO_2_ emissions.

In summary, the *ZEYs* obtained here can prove useful for answering three simple questions related to the climate change issue: what to do (eliminate CO_2_ emissions), when (between 2030 and 2050), and who (the current generation).

### Personal quota and personal behaviour

In this article, we showed that 5.0 tons of CO_2_ yr^-1^ p^-1^ is a representative value for past and future levels in per-capita emissions of CO_2_ (Fig 6), and we defined this value as the personal quota. First, Fig 6 shows that world average per-capita emissions have been roughly constant since 1960, suggesting the important role of the human population in the increase of CO_2_ emissions.

The goal of this work is to help individuals with the “grasping problem” of the climate change issue, where the definition of the personal quota and *ZEY* should facilitate strategies to encourage pro-environmental actions. Previously mentioned strategies include reducing cognitive effort and taking advantage of social influence [42]. Consequently, these emissions boundaries are expressed in an easily readable format. For example, to reduce cognitive barriers, the results of this study can be easily communicated: “between 2030 and 2050, anthropogenic CO_2_ emissions should be nearly zero” or more assertively “by 2030, anthropogenic CO_2_ emissions need to be eliminated” [43]. Furthermore, pro-environmental actions are more likely when collective associated costs and benefits are considered [42]; in this context, the existence of a uniform personal quota that reinforces the fact that climate change is attributable to the collective action of billions of people, helps individuals both to understand the climate change problem and to see the solution of a global matter from a personal perspective. Hence, with the definition of the personal quota of CO_2_ emissions, we are proposing that individuals reinforce their understanding about boundaries to personal activities linked to greenhouse gas emissions.

Furthermore, the definition of a personal quota can help evaluate measures that can be taken individually to achieve global targets. Based on the personal quota, carbon footprint calculators could be used to measure and manage personal emissions related to food, transportation and goods (furniture, car or electronic devices, for instance) to meet the personal quota of 5.0 tons of CO_2_ yr^-1^ p^-1^. Examples of CO_2_ emissions as a function of specific actions are given in Table 1.

**Table 1:** Examples of CO_2_ emissions as a function of specific actions. Values are presented in terms of net emission and percentages of the personal quota.

Action	Emissions	% of personal annual quota	Reference
Filling up a car with a 50 L gas tank with fuel once a week	6720 kg CO_2_	134%	[44]
Consumption of one kg of meat per week Beef Pig Chicken	696 kg CO_2_ 169 kg CO_2_ 187 kg CO_2_	13.9% 3.3% 3.7%	[45]
Buying a new car	4.9 ton CO_2_ 28.7 ton CO_2_	98% 574%	Examples from [41], using the transformation between CO_2e_ and CO_2_ of Fig 3
Buying a new computer	164 kg CO_2_ 656 kg CO_2_	3.3% 13.1%	
London to Glasgow (556 km) and return Plane Train	410 kg CO_2_ 98.4 kg CO_2_	8.2% 2.0%	
Flying from London to Hong Kong	3.77 ton CO_2_	75.4%	
Insulating an apartment Payback over 40 years	287 kg CO_2_ -28.7 ton CO_2_	5.7% -574%	

Likewise, the definition of the personal quota sends a clear message about the remaining budget of greenhouse gas emissions that would enable the achievement of the “well below 2°C” carbon limit.

The proposed definition of a personal quota and a *ZEY* contributes to the burden-sharing frameworks for individuals to solve the climate change problem. In this sense, it shares the definition of fairness that two persons who emit the same amount of CO_2_ should equally contribute to reducing CO_2_ emissions, regardless where they live [29]. It also helps people to visualise the link between personal actions and the planetary global budget of CO_2_. The issue of homogeneous personal quota has been a subject of debate [28,46], because it does not consider past responsibilities, it could be read as a right to pollute, and it does not consider that the costs of mitigation and damages are not homogeneous [47]. However, it is argued that a uniform personal quota reveals both “the common but differentiated responsibility” and the “respective capabilities” principles as it puts pressure on today’s highly emitter individuals, which on average are also individuals with higher incomes given the positive correlation between personal income and CO_2_ emissions [9,13,29]. A uniform personal quota should not be interpreted as a “right to emit” more CO2 for citizens with emissions below 5.0 tons of CO_2_ yr^-1^ p^-1^. Such an interpretation does not consider that global warming primarily depends on the total mass of emitted CO_2_, and therefore any increase in today’s per capita emissions only worsens the climate change problem and increase future mitigation costs.

In summary, this work’s primary contribution is to connect an equal per capita fairness approach to a global carbon budget, by linking personal levels with planetary levels through the definition of two numbers in an easily readable format. The *ZEY* (between 2030 and 2050) and personal quota (5.0 tons of CO_2_ yr^-1^ p^-1^), are expected to help individuals to more easily comprehend and take action in regards to the climate change issue. However, the sooner the world average per-capita emissions start to decrease, the longer the available time to eliminate net CO_2_ emissions.

## Supporting information

S1 Appendix Compressed file containing the input files (*.SCEN) of the different scenarios used for validating the ZEY and personal quota with model MAGICC (http://live.magicc.org/). (ZIP)

## References

[1] Stocker TF. The Closing Door of Climate Targets. Science (80-). 2013;339: 280–282. doi: 10.1126/science.1232468 23196911 10.1126/science.1232468

[2] Meinshausen M, Meinshausen N, Hare W, Raper SCB, Frieler K, Knutti R, et al Greenhouse-gas emission targets for limiting global warming to 2°C. Nature. Nature Publishing Group; 2009;458: 1158–1162. doi: 10.1038/nature08017 19407799 10.1038/nature08017

[3] Friedlingstein P, Andrew RM, Rogelj J, Peters GP, Canadell JG, Knutti R, et al Persistent growth of CO2 emissions and implications for reaching climate targets. Nat Geosci. Nature Research; 2014;7: 709–715. doi: 10.1038/ngeo2248

[4] Collins M, Knutti R, Arblaster J, Dufresne J-L, Fichefet T, Friedlingstein P, et al Chapter 12: Long-term Climate Change: Projections, Commitments and Irreversibility IPCC AR5 WG1 2013. Cambridge University Press; 2013 pp. 1029–1136.

[5] UNFCCC. Conference of the Parties (COP). Adoption of the Paris Agreement. Proposal by the President. Paris Climate Change Conference—November 2015, COP 21. 2015. p. 32. FCCC/CP/2015/L.9/Rev.1

[6] Matthews HD, Caldeira K. Stabilizing climate requires near-zero emissions. Geophys Res Lett. 2008;35: L04705 doi: 10.1029/2007GL032388

[7] IPCC. Synthesis Report. Contribution of Working Groups I, II and III to the Fifth Assessment Report of the Intergovernmental Panel on Climate. Geneva, Switzerland: IPCC; 2014.

[8] Knutti R, Rogelj J. The legacy of our CO2 emissions: a clash of scientific facts, politics and ethics. Clim Change. Springer Netherlands; 2015;133: 361–373. doi: 10.1007/s10584-015-1340-3

[9] Bear P, Harte J, Haya B, Herzog A V., Holdren J, Hultman NE, et al Equity and Green house gas responsibility. Science (80-). 2000;289: 2287.

[10] Kintisch E. Climate change. Deforestation moves to the fore in Copenhagen. Science (New York, N.Y.). 2009 p. 1465 20007869 10.1126/science.326.5959.1465

[11] Raupach MR, Davis SJ, Peters GP, Andrew RM, Canadell JG, Ciais P, et al Sharing a quota on cumulative carbon emissions. Nat Clim Chang. Nature Research; 2014;4: 873–879. doi: 10.1038/nclimate2384

[12] van Renssen S. Getting a fair deal. Nat Clim Chang. Nature Research; 2015;5: 513–514. doi: 10.1038/nclimate2661

[13] Baer P. The greenhouse development rights framework for global burden sharing: Reflection on principles and prospects. Wiley Interdiscip Rev Clim Chang. John Wiley & Sons, Inc.; 2013;4: 61–71. doi: 10.1002/wcc.201

[14] Meinshausen M, Jeffery L, Guetschow J, Robiou du Pont Y, Rogelj J, Schaeffer M, et al National post-2020 greenhouse gas targets and diversity-aware leadership. Nat Clim Chang. Nature Research; 2015;5: 1098–1106. doi: 10.1038/nclimate2826

[15] IPCC. Summary for Policymakers. In: Climate Change 2014: Mitigation of Climate Change. Contribution of Working Group III to the Fifth Assessment Report of the Intergovernmental Panel on Climate Change. Cambridge: Cambridge University Press; 2014.

[16] Hoekstra AY, Wiedmann TO. Humanity’s unsustainable environmental footprint. Science (80-). 2014;344: 1114–1117. doi: 10.1126/science.1248365 24904155 10.1126/science.1248365

[17] Corner A, Roberts O, Chiari S, Völler S, Mayrhuber ES, Mandl S, et al How do young people engage with climate change? The role of knowledge, values, message framing, and trusted communicators. Wiley Interdiscip Rev Clim Chang. John Wiley & Sons, Inc.; 2015;6: 523–534. doi: 10.1002/wcc.353

[18] Palmer C. Three Questions on Climate Change. Ethics Int Aff. Cambridge University Press; 2014;28: 343–350. doi: 10.1017/S0892679414000410

[19] Hardin GLB-H. The Tragedy of the Commons. Science (80-). 1968;162: 1243–1248. 5699198

[20] Howell RA. It’s not (just) “the environment, stupid!” Values, motivations, and routes to engagement of people adopting lower-carbon lifestyles. Glob Environ Chang. 2013;23: 281–290. doi: 10.1016/j.gloenvcha.2012.10.015

[21] Broomell SB, Budescu D V., Por H-H. Personal experience with climate change predicts intentions to act. Glob Environ Chang. 2015;32: 67–73. doi: 10.1016/j.gloenvcha.2015.03.001

[22] Blennow K, Persson J, Tomé M, Hanewinkel M, Moser S, Ekstrom J, et al Climate Change: Believing and Seeing Implies Adapting. Krueger F, editor. PLoS One. Public Library of Science; 2012;7: e50182 doi: 10.1371/journal.pone.0050182 23185568 10.1371/journal.pone.0050182 PMC3504002

[23] Lee TM, Markowitz EM, Howe PD, Ko C-Y, Leiserowitz AA. Predictors of public climate change awareness and risk perception around the world. Nat Clim Chang. Nature Research; 2015;5: 1014–1020. doi: 10.1038/nclimate2728

[24] Fishbein M, Ajzen I. Belief, Attitude, Intention, and Behavior: An Introduction to Theory and Research. Addison-Wesley Addison-Wesley; 1975.

[25] Max-neef M a., Hopenhayn M, Hamrell S. Human Scale Development: Conception, Application and Further Reflections, Volume 1 New York and London: The Apex Press; 1991.

[26] Janerio R De. United Nations Conference on Environment & Development Rio de Janerio, Brazil, 3 to 14 June 1992. Reproduction. 1992; 351. 10.1007/s11671-008-9208-3

[27] Agarwal A, Narain S. Global Warming in an Unequal World: A Case of Environmental Colonialism. New Dehli, India: Centre for Science and Environment;

[28] WBGU. Solving the climate dilemma: The budget approach. Special Report. Special Report., editor. Berlin: German Advisory Council on Global Change. Berlin; 2009.

[29] Chakravarty S, Chikkatur A, de Coninck H, Pacala S, Socolow R, Tavoni M. Sharing global CO2 emission reductions among one billion high emitters. Proc Natl Acad Sci U S A. National Academy of Sciences; 2009;106: 11884–8. doi: 10.1073/pnas.0905232106 19581586 10.1073/pnas.0905232106 PMC2706272

[30] Taylor KE, Stouffer RJ, Meehl GA. An overview of CMIP5 and the experiment design. Bulletin of the American Meteorological Society. 2012 pp. 485–498.

[31] Meinshausen M, Raper SCB, Wigley TML. Emulating coupled atmosphere-ocean and carbon cycle models with a simpler model, MAGICC6—Part 1: Model description and calibration. Atmos Chem Phys. 2011;11: 1417–1456.

[32] Rogelj J, Hare W, Lowe J, van Vuuren DP, Riahi K, Matthews B, et al Emission pathways consistent with a 2°C global temperature limit. Nat Clim Chang. Nature Research; 2011;1: 413–418. doi: 10.1038/nclimate1258

[33] IPCC. Annex I: Atlas of Global and Regional Climate Projections. In: Climate Change 2013: The Physical Science Basis. Contribution of Working Group I to the Fifth Assessment Report of the Intergovernmental Panel on Climate Change. Cambridge: Cambridge University Press; 2013.

[34] van Vuuren DP, Edmonds J, Kainuma M, Riahi K, Thomson A, Hibbard K, et al The representative concentration pathways: An overview. Clim Change. 2011;109: 5–31.

[35] Rogelj J, Schaeffer M, Meinshausen M, Knutti R, Alcamo J, Riahi K, et al Zero emission targets as long-term global goals for climate protection. Environ Res Lett. 2015;10: 105007 Available: http://stacks.iop.org/1748-9326/10/i=10/a=105007

[36] Rogelj J, Schaeffer M, Friedlingstein P, Gillett NP, van Vuuren DP, Riahi K, et al Differences between carbon budget estimates unravelled. Nat Clim Chang. Nature Research; 2016;6: 245–252. doi: 10.1038/nclimate2868

[37] Le Quéré C, Andrew RM, Canadell JG, Sitch S, Ivar Korsbakken J, Peters GP, et al Global Carbon Budget 2016. Earth Syst Sci Data. Copernicus GmbH; 2016;8: 605–649. doi: 10.5194/essd-8-605-2016

[38] Tebaldi C, Knutti R. The use of the multi-model ensemble in probabilistic climate projections. Philos Trans R Soc London A Math Phys Eng Sci. 2007;365. 10.1098/rsta.2007.2076 17569654

[39] Collins M, Knutti R, Arblaster J, Dufresne J-L, Fichefet T, Friedlingstein P, et al. Climate Change 2013: The Physical Science Basis. Contribution of Working Group I to the Fifth Assessment Report of the Intergovernmental Panel on Climate Change, ed T F Stocker, D. Qin, G.-K. Plattner, M. Tignor, S.K. Allen, J. Boschung, A. Nauels, Y. Xia. Stocker TF, Qin D, Plattner G-K, Tignor M, Allen SK, Boschung J, et al., editors. Cambridge, United Kingdom and New York, NY, USA: Cambridge University Press; 2013.

[40] Nations U. World Population Prospects: The 2015 Revision. United Nations Econ Soc Aff. 2015;XXXIII: 1–66.

[41] Berners-Lee M. How bad are bananas?: The Carbon Footprint of Everything. Profile Books Plt; 2010.

[42] Steg L. Values, Norms, and Intrinsic Motivation to Act Proenvironmentally. Annu Rev Environ Resour. Annual Reviews; 2016;41: 277–292. doi: 10.1146/annurev-environ-110615-085947

[43] Kronrod A, Grinstein A, Wathieu L. Go Green! Should Environmental Messages Be So Assertive? J Mark. 2012;76: 95–102. doi: 10.1509/jm.10.0416

[44] Ipcc. 2006 IPCC Guidelines for National Greenhouse Gas Inventories, Prepared by the National Greenhouse Gas Inventories Programme [Internet]. Agriculture Forestry and Other Land Use. 2006. http://www.ipcc-nggip.iges.or.jp/public/2006gl/pdf/2_Volume2/V2_3_Ch3_Mobile_Combustion.pdf

[45] Gerber P.J., Steinfeld H., Henderson B., Mottet A., Opio C., Dijkman J., Falcucci A. & Tempio G. Tackling climate change through livestock—A global assessment of emissions and mitigation opportunities.Food and Agriculture Organization of the United Nations (FAO), Rome 2013.

[46] Schuppert F, Seidel C. Equality, justice and feasibility: an ethical analysis of the WBGU’s budget approach. Clim Change. Springer Netherlands; 2015;133: 397–406.

[47] Matthews HD. Quantifying historical carbon and climate debts among nations. Nat Clim Chang. Nature Research; 2015;6: 60–64. doi: 10.1038/nclimate2774

